# 

**Published:** 1883-07

**Authors:** 


JULY, 1883.

Vol. I.] [No. 1.

### THE BRISTOL MEDICO-CHIRURGICAL JOURNAL

A Journal of the Medical Sciences for the West of England and South Wales

*PUBLISHED UNDER THE AUSPICES OF*

*THE BRISTOL MEDICO-CHIRURGICAL SOCIETY*

EDITED BY

J. GREIG SMITH

M.A., F.R.S.E.

INDEX MEDICUS.

"Scire est nescire, nisi id me

Scire alius sciret."

[Half-Yearly.]

BRISTOL: J. W. ARROWSMITH

LONDON: J. & A. CHURCHILL, 11 NEW BURLINGTON STREET

*Price Two Shillings and Sixpence.*

